# Cutaneous diphtheria: three case-reports to discuss determinants of re-emergence in resource-rich settings

**DOI:** 10.1080/22221751.2021.2008774

**Published:** 2021-12-06

**Authors:** Laura I. Levi, Frédéric Barbut, Dorothée Chopin, Paul Rondeau, Valérie Lalande, Sarah Jolivet, Edgar Badell, Sylvain Brisse, Karine Lacombe, Laure Surgers

**Affiliations:** a Service des Maladies Infectieuses et Tropicales, Hôpital Saint-Antoine, GHU APHP. Sorbonne Université, Paris, France; b Unité de Prévention du Risque Infectieux, hôpital Saint-Antoine, GHU APHP. Sorbonne Université, Paris, France; c Département de bactériologie, hôpital Saint-Antoine, GHU APHP. Sorbonne Université, Paris, France; d Biodiversity and Epidemiology of Bacterial Pathogens, Institut Pasteur, Paris, France; e National Reference Center for Corynebacteria of the Diphtheriae Complex, Institut Pasteur, Paris, France; f Sorbonne Université, INSERM, Institut Pierre Louis d'Épidémiologie et de Santé Publique, Paris, France

**Keywords:** Diphtheria, *Corynebacterium diphtheriae*, *Corynebacterium ulcerans*, emergence, vaccine scepticism

## Abstract

Diphtheria is a re-emerging disease in resource-rich settings. We here report three cases of cutaneous diphtheria diagnosed and managed in our infectious disease department and discuss the determinants of its re-emergence. Migration, travel and vaccine scepticism are key factors not only for diphtheria re-emergence, but for the future of most preventable diseases.

Dear Editor,

Diphtheria is a re-emerging health threat in multiple resource-rich settings. After many years with very few cases, several countries in Europe and North America have detected a significant increase in diphtheria infections during the last decade, including respiratory and cutaneous diphtheria [1–4], as reported by the European centre for disease prevention and control (ECDC) [5]. Since 2018, we have treated three cases of cutaneous diphtheria in an infectious diseases department set in a large University teaching hospital.

The first patient was a 16-year-old Afghan refugee who arrived in France one month earlier through the overland route. He presented at the hospital for a sore throat and a left hallux paronychia that was surgically excised. He started an antibiotic course with amoxicillin and clavulanate acid before surgical sampling of the paronychia revealed a week later a coinfection with methicillin-sensitive *Staphylococcus aureus*, *Streptococcus pyogenes* and toxigenic *Corynebacterium diphtheriae* (Elek positive). A throat swab performed after six days of antibiotherapy came negative for *C. diphtheriae*. During hospitalization, no toxigenic complication was noted. Anti-toxin serum was well tolerated, and antibiotherapy was switched to amoxicillin alone for a total duration of 14 days. His vaccination status was unknown.

The second patient was a 33-year-old man, born in France from Malian origins without any previous medical history. He travelled to Mali for 3 weeks both in urban and rural areas where he suffered a motorbike accident leading to limb wounds. Two days after he returned to France, the patient presented at the hospital because the wounds did not heal after three weeks of evolution. Examination showed clean superficial ulcerations and no local sampling was performed. Tetanus quick test was positive (he was correctly vaccinated against tetanus diphtheria and poliomyelitis during childhood but did not get his 25-year-old booster) and he was prescribed simple local cares. Ten days later, the patient injured his left index finger creating a painful paronychia (Figure 1) that was surgically excised. Surgical samples identified *S. pyogenes* and toxigenic *C. diphtheriae* (Elek positive) leading to a 14-day course of amoxicillin and anti-toxin serum infusion. He did not present any systemic complications of diphtheria. Throat swabs confirmed pharyngeal carriage of *C. diphtheriae* and 3-day azithromycin treatment was added.

**Figure 1.**
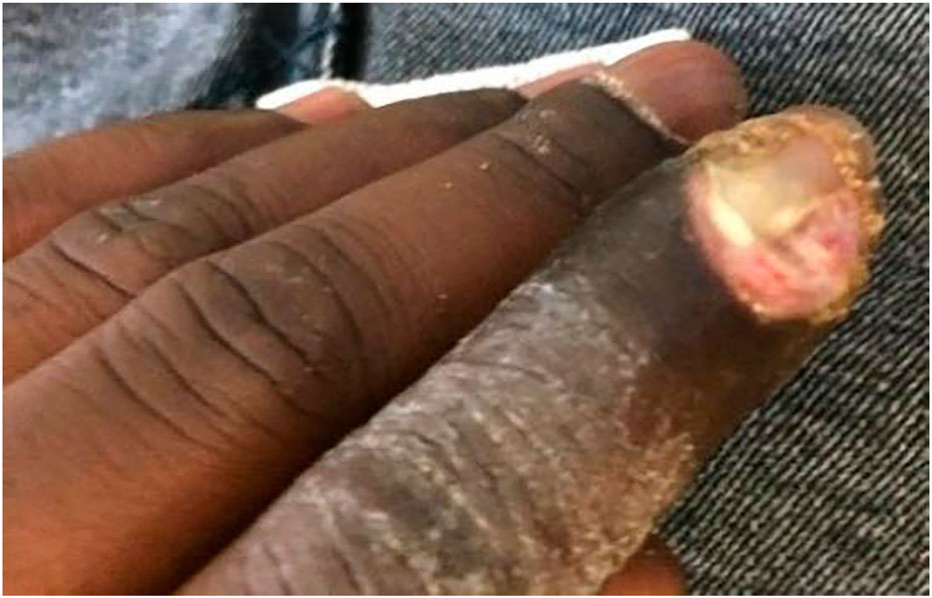
Cutaneous diphtheria in the form of a paronychia appearing after a travel to Mali.

The third patient was a 52-year-old woman, living with many pets (cats, dogs, rats and hamsters), whose main medical history was self-inflicted cutaneous lesions leading to several surgical interventions including amputation upstream her right wrist and many antibiotic treatments. Because of a new ulceration on her amputation stump, she got a cutaneous swab revealing infection with methicillin-sensitive *S. aureus* and toxigenic *Corynebacterium ulcerans* (Elek positive, sequence type ST 332). A throat swab revealed pharyngeal carriage of the same strain of *C. ulcerans* (without any respiratory or systemic symptoms) and a 3-day azithromycin treatment was added to the 14-day course of amoxicillin and local cares. The patient had not been vaccinated against diphtheria for more than 30 years.

For all three patients, post-treatment control throat swabs cultures came negative, and local wounds evolved favourably, even though the third patient needed an extra 2 week-course of amoxicillin to treat cutaneous carriage. Their vaccinations were updated according to French guidelines. Diphtheria was declared to the Health Authorities and to the Infection Control Unit, while investigations were performed to identify, treat and vaccinate their contacts.

*C. diphtheriae* and *C. ulcerans* are the main causative agent of diphtheria [5–7]. Some strains are able to secrete the diphtheria toxin responsible for myocardial and neurological complications, leading to the high mortality rate observed for classical diphtheria. Although this form of infection is the most threatening one, cases of cutaneous infections or colonization have been recently reported. Cutaneous forms are nonspecific and usually develop on limbs after wounds. Classical features include one or several well-delimitated ulcers classically covered with pseudo-membranes. Microbiological diagnosis relies on the identification of *C. diphtheriae* or *ulcerans* in culture from cutaneous swabs, often associated with *S. pyogenes* and *S. aureus* [4], such as in our cases. Both toxigenic and non-toxigenic strains can cause cutaneous infection, but resulting systemic complications from toxigenic strains via this route are rare [4]. Since *C. diphtheriae* is naturally susceptible to amoxicillin that is the usual treatment for cutaneous infections, cutaneous diphtheria may be cured before determination of the possible toxigenic nature of the agent. This has led several authors to question the need for anti-toxin serotherapy in association with antibiotics in cutaneous forms with toxigenic strains [4]. This is particularly relevant as anti-toxin serum is difficult to obtain and involves a high risk of severe anaphylaxis [4,6,7]. Hence, World Health Organization and Public Health England do not recommend it anymore [6,7], but following French guidelines, in which it is still recommended [8,9], patients 1 and 2 received anti-toxin, contrarily to patient 3. Furthermore, anti-diphtheria vaccine prevents toxigenic complications of the disease but not the carriage or the infection itself which can still occur such as for the second patient [4–6].

Migration, poverty and geopolitical conflicts especially in the poor hygiene refugee camp, may favour re-emergence of diphtheria, and more precisely its cutaneous form [1,10,11], as reflected by the Afghan patient. He might have been infected in his home country where vaccination coverage is low (66% of children received 3 doses of the vaccine in 2016) [12] or in refugee camps during his travel to France [10,11]. Other key factors include globalization and travel, as for the second patient who was born in France but originated from a high prevalence country and might have been infected during a travel there, considering the low vaccination coverage of Mali (76% in 2016) compared to France (96% in 2016) [12]. Finally, the constant decrease of vaccination coverage over time plays an important role [2], and favour indigenous infection with a risk of toxigenic infection as for the third patient who never left France and probably got infected through pet contact [3,6]. The example of France is a telling one since it is the European country with the highest vaccine scepticism [13]. According to ECDC, a decrease in adult booster coverage might explain re-emergence of the toxigenic strains in European countries [4,5]. Hence, the French reference centre received 163 clinical isolates between 2008 and 2017, with a third of toxicogenic strains [14]. Interestingly, for all patients infected outside or inside France, and prior to the diagnosis of diphtheria, there is the possibility of transmission to close contacts, with a risk of toxigenic symptoms, in the absence of protective antibodies from vaccination. In the light of such examples, preventing re-emergence of toxigenic diphtheria or any vaccine-preventable disease should be an additional reason to promote vaccine policies worldwide.
